# Outcome measures used in trials on gait rehabilitation in multiple sclerosis: A systematic literature review

**DOI:** 10.1371/journal.pone.0257809

**Published:** 2021-09-30

**Authors:** L. Santisteban, M. Teremetz, J. Irazusta, P. G. Lindberg, A. Rodriguez-Larrad

**Affiliations:** 1 Department of Physiology, University of the Basque Country, UPV/EHU, Leioa, Spain; 2 Biocruces Bizkaia Health Research Institute, Barakaldo, Bizkaia, Spain; 3 Institute of Psychiatry and Neuroscience of Paris, INSERM U1266, Université Paris Descartes, Sorbonne Paris Cité, Paris, France; BG-Universitatsklinikum Bergmannsheil, Ruhr-Universitat Bochum, GERMANY

## Abstract

**Background:**

Multiple Sclerosis (MS) is associated with impaired gait and a growing number of clinical trials have investigated efficacy of various interventions. Choice of outcome measures is crucial in determining efficiency of interventions. However, it remains unclear whether there is consensus on which outcome measures to use in gait intervention studies in MS.

**Objective:**

We aimed to identify the commonly selected outcome measures in randomized controlled trials (RCTs) on gait rehabilitation interventions in people with MS. Additional aims were to identify which of the domains of the International Classification of Functioning, Disability and Health (ICF) are the most studied and to characterize how outcome measures are combined and adapted to MS severity.

**Methods:**

Pubmed, Cochrane Central, Embase and Scopus databases were searched for RCT studies on gait interventions in people living with MS according to PRISMA guidelines.

**Results:**

In 46 RCTs, we identified 69 different outcome measures. The most used outcome measures were 6-minute walking test and the Timed Up and Go test, used in 37% of the analyzed studies. They were followed by gait spatiotemporal parameters (35%) most often used to inform on gait speed, cadence, and step length. Fatigue was measured in 39% of studies. Participation was assessed in 50% of studies, albeit with a wide variety of scales. Only 39% of studies included measures covering all ICF levels, and Participation measures were rarely combined with gait spatiotemporal parameters (only two studies).

**Conclusions:**

Selection of outcome measures remains heterogenous in RCTs on gait rehabilitation interventions in MS. However, there is a growing consensus on the need for quantitative gait spatiotemporal parameter measures combined with clinical assessments of gait, balance, and mobility in RCTs on gait interventions in MS. Future RCTs should incorporate measures of fatigue and measures from Participation domain of ICF to provide comprehensive evaluation of trial efficacy across all levels of functioning.

## 1. Introduction

### 1.1. Rationale

Multiple Sclerosis (MS) is an inflammatory demyelinating chronic disease of the central nervous system, and it is the most common non-traumatic cause of disability among young adults [1]. The clinical presentation and evolution of this disease is very heterogeneous, generating quite different disorders with important functional repercussions [2]. Gait impairment is one of the most common motor disorders [3] and is perceived as one of the most important bodily functions across the MS disability spectrum [4].

There is a central nervous system remodeling after inflammatory and demyelinating injuries by spontaneous mechanisms of recovery [1] that can be enhanced by rehabilitation interventions that promote activity dependent neural plasticity [5], improve the degree of functionality and increase Participation [6, 7].

In recent years, with advances in the field of technology and neurorehabilitation, there have been a growing number of new rehabilitation approaches and RCTs to assess their efficacy [8]. Assessment in this context is central and selecting the most appropriate outcome measures is crucial for determining which rehabilitation treatments are most efficient [9]. There are many assessment tools, clinical scales, self-questionnaires, and technological devices that are validated and commonly used in gait assessment in MS [8, 10]. Psychometric properties of some of these assessment methods have already been studied by many authors [11, 12]. However, a consensus about which are the most appropriate is lacking, although agreement is crucial to generalize outcomes.

Primary symptoms of MS impact not only on disability and functioning but can also have major effects on quality of life and socioeconomic issues. The World Health Organization proposes a framework and classification for measuring health and disability known as the International Classification of Functioning, Disability and Health (ICF). According to the ICF, health domains of people living with MS (pwMS) are classified into three levels: Body structure/Body function, Activity, and Participation domains [13, 14]. In RCTs, assessing health according to all three ICF domains is considered beneficial in determining efficacy of rehabilitation techniques in the different health domains. For example, including a measure from the Participation domain would provide information on whether the bio-psycho-social situation of people changes following the rehabilitation intervention. Gait rehabilitation interventions can improve not only walking abilities, classified in the ICF Activity domain, but also other aspects like strength, range of movement or spasticity, included in the Body function/Body structure domain, and aspects like self-esteem, social interaction or quality of life, included in the ICF Participation domain [10, 15].

European Multiple Sclerosis rehabilitation recommendations [16] state that a comprehensive view of the pwMS status across all ICF domains is needed to provide adequate health care. It is emphasized to select outcome measures according to the ICF framework in clinical trials on MS rehabilitation.

There is a need for a systematic literature review focusing on assessment methods used in clinical trials on gait rehabilitation interventions in pwMS in recent years. This would inform on which outcome measures are most used in the clinical and scientific community. If measures are quite common across all studies, this would indicate a good consensus in the field. Knowing which outcome measures are used in clinical trials is a first step that would help improve the design of future studies by identifying weaknesses and strong points in gait assessment procedures.

The first aim of this systematic review was to identify the commonly selected outcome measures in randomized controlled trials (RCTs) on gait rehabilitation interventions in pwMS.

Secondary aims were to identify which of the domains of the ICF are the most studied and to characterize how outcome measures are combined and adapted to MS severity.

## 2. Methods

### 2.1. Study design and search strategy

A systematic literature review was performed according to PRISMA guidelines 2009 [17] and following the recommendations provided in the Cochrane handbook for literature reviews [18].

The search was performed in the following databases: Medline using Pubmed interface, Cochrane Central, Embase and Scopus.

The search strategy included articles from January 2010 until February 2021, using the following key words and Mesh terms: ("Walking"[Mesh] OR "Gait"[Mesh] OR "Gait Disorders, Neurologic"[Mesh] OR "Mobility Limitation"[Mesh]) AND ("Rehabilitation"[Mesh] OR "rehabilitation" [Subheading] OR "Physical and Rehabilitation Medicine"[Mesh] OR "Neurological Rehabilitation"[Mesh] OR "Exercise Therapy"[Mesh]) AND (“Multiple Sclerosis"[Mesh]).

The literature search included manual scanning of the reference lists of the included articles.

We limited the search (using database filters) to studies performed on human adults and published from 1/1/2010 to 28/02/2021.

Two independent reviewers (L.S., A.R.-L.) identified which articles to include.

The search and selection processes were performed independently by both L.S. and A.R.-L. Disagreements on whether to include a study were resolved by discussing with a third author (J.I) and reaching consensus.

### 2.2. Study identification

Following the removal of duplicates with Refworks and verifying them manually, included studies were identified by first screening the title and abstract and, secondly, by full text screening.

Articles were included if they fulfilled the following inclusion criteria: i) randomized clinical trials regarding rehabilitation interventions to improve gait capacities in pwMS, ii) adult participants > 18 years old. Exclusion criteria included: i) literature reviews, ii) study protocols, iii) studies regarding the psychometric properties of outcome measures, iv) studies combining participants with other neurological diseases, v) studies evaluating specific rehabilitation interventions of other impairments (e.g. upper limb rehabilitation interventions, pelvic floor muscle rehabilitation interventions, memory rehabilitation interventions, swallowing rehabilitation interventions, balance specific rehabilitation interventions, vestibular rehabilitation interventions), if the aim of the intervention was not to improve gait capacities.

### 2.3. Data extraction

Full articles were reviewed for: year of publication, characteristics of the participants (age, disease severity according to EDSS, form of MS), type of rehabilitation intervention, number of participants and reported outcome measures.

### 2.4. Data analysis

The data have been analyzed using Microsoft Excel software. Figs 2 and 3 were created with Excel software and Fig 4 with Gimp software.

Data are available.

## 3. Results

The electronic search yielded 88 articles in Pubmed, 90 in Cochrane Central, 363 in Embase and 258 in Scopus.

**Fig 1:**
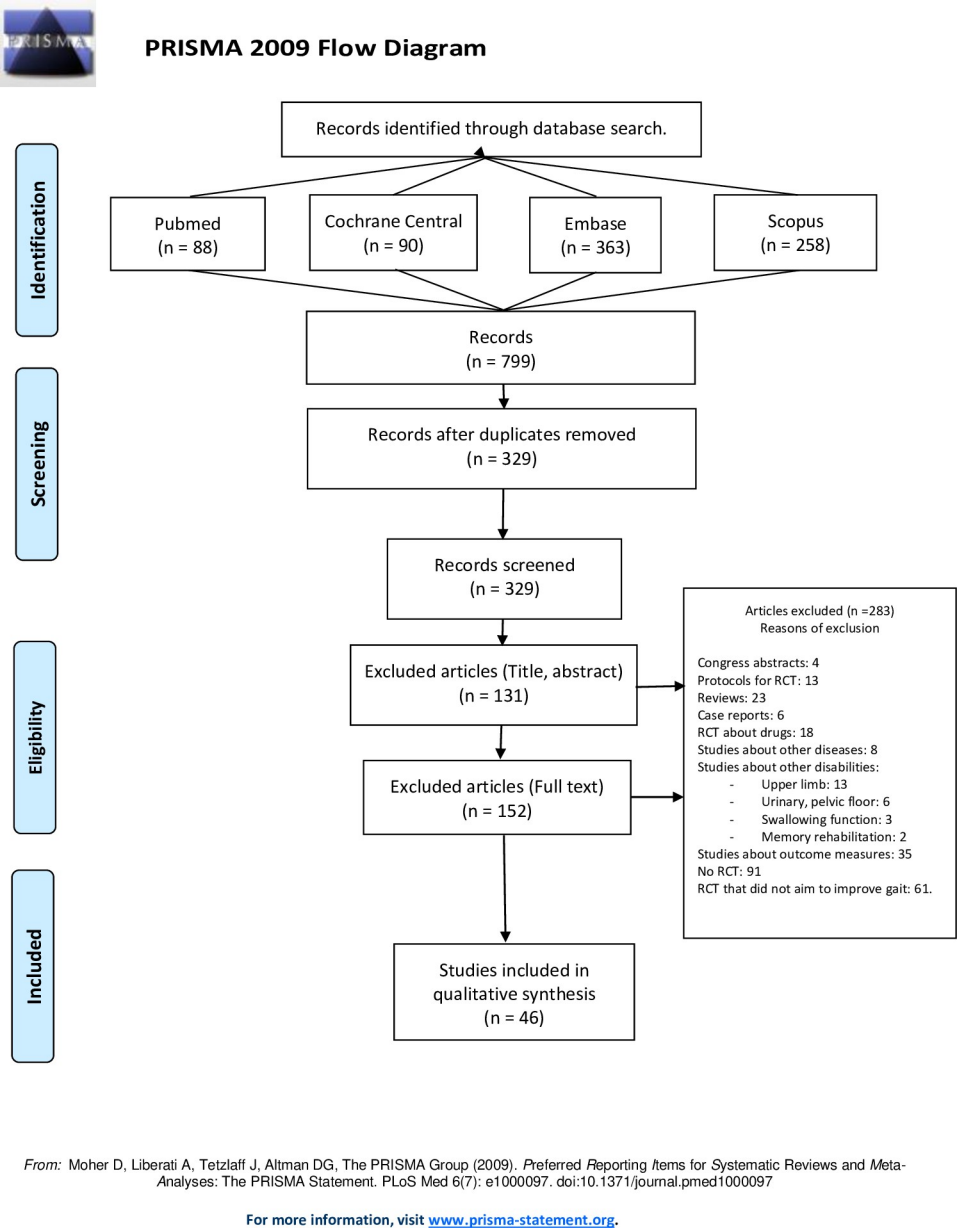
Flow chart of the methodology for study selection.

The selection process is explained in Fig 1.

Forty-six articles [19–64] shown in Table 1. fulfilled selection criteria, involving a total of 1842 patients. 69 outcome measures were identified in included RCTs, they are shown in Table 2. The summary of data collection is shown in Table 1.

**Table 1:** Summary of analyzed articles.

Article	Participant characteristics: sample size (N), disease severity (mean/range; EDSS/PDSS), age (mean years), MS type	Intervention	Assessments
**Martini et al., 2018** [19]	N 40; EDSS:6; Age 56	Multicomponent walking aid program	TUG, 2MWT, T25W, FSST, MSWS-12, MSIS-29, ABC
	MS type: NR		
**Russo et al., 2017** [20]	N 45; EDSS:3–5.5; Age 42	Robot gait training with virtual reality (Lokomat)	TUG, EDSS, TBS, HRSD
	MS type: RR 45		
**Sandroff et al., 2017** [21]	N 83; PDDS:4; Age 49.8	Multimodal exercise training	GSTP, VO2, MS-IM, T25W, 6MWT, MSWS-12
	MS type: NR		
**Calabro et al., 2017** [22]	N 40; EDSS: 4.1–5.5; Age 41	Robot assisted therapy with virtual reality	MAS, MS-LD, TUG, BBS, HRSD, COPE
	MS type: NR		
**Conroy et al., 2017** [23]	N 24; PDDS:4.4; Age 50.4	Self-directed exercise at home	T25W, BBS, 6MWT, MSWS-12
	MS type: RR8, SP15, PP1		
**Pau et al.,**	N 22; EDSS: 3.6; Age 47.4	Adapted physical activities	ROM, GSTP
**2017** [24]	MS type: NR		
**Pompa et al., 2016** [25]	N 43; EDSS: 6–7.5; Age 47	Robot assisted gait training + conventional rehabilitation	VAS, FSS, EDSS, 2MWT, FAC
	MS type: SP40, PP3		
**Davies et al.,**	N 32; EDSS: 5.4; Age 53.3	High frequency physical therapy	GSTP, 6MWT
**2016** [26]	MS type: RR22, SP10		
**Kalron et al., 2016** [27]	N 45; EDSS:4.6; Age 44.3	Pilates/physical therapy for improving gait	FIS, TUG, 2MWT, FSST, BBS, 6MWT, MSWT-12
	MS type: RR45		
**Straudi et al., 2016** [28]	N 52; EDSS:6.43; Age 52.3	Robot assisted gait training	FSS, TUG, BBS, 6MWT, 10m, QoLSF-36, PHQ-9
	MS type: SP36, PP16		
**Straudi et al., 2014** [29]	N 24; EDSS:4.89; Age 52.6	Task oriented circuit training	FSS, TUG, 6MWT, DGI, MSWS-12, MSIS-29
	MS type RR6, SP8, PP10		
**Tyler et al., 2014** [30]	N 20; EDSS:5.25; Age:55.4	Non-invasive neuromodulation/physical therapy	DGI
	MS type: RR13, SP6, PP1		
**Aydin et al., 2014** [31]	N 40; EDSS:3.6; Age 33	Home based calisthenic exercises	FSS, BBS, 10m, HADS, MusiQoL
	MS type: RR40		
**Ruiz et al., 2013** [32]	N 7; EDSS: 5; Age 47	Robot assisted, body weight support training	GSTP, T25W, FRT, 6MWT
	MS type: RR5, PP2		
**Schwartz et al., 2012** [33]	N 32; EDSS: 6; Age 48.6	Robot assisted gait training	TUG, EDSS, BBS, 6MWT, 10m, Rand-36
	MS type: NR		
**Vaney et al., 2012** [34]	N 49; EDSS:5.8; Age 56.2	Robot assisted step training (Lokomat)	MAS, VAS, BBS, 3MWT, 10m, Ac, WE
	MS type: NR		
**Dodd et al.,**	N 71; EDSS:0–6.5; Age49	Progressive resistance training	MSSS-88, MS-MD, FIS,2MWT, WHOQoL-Bref
**2011** [35]	MS type: RR71		
**Conklyn et al., 2010** [36]	N 10; EDSS NR Age: 48.5	Home based walking program (rhythmic auditory)	MAS, VAS, MRC, Kinematics, PDDS, T25W, AI
	MS type: RR7, SP2, PP1		
**Cakit et al., 2010** [37]	N 45; EDSS:0–6; Age 38.1	Cycling progressive resistance training programs	FSS, TUG, FRT, 10m, DGI, FES, QoLSF-36, BDI
	MS type: NR		
**Robinson et al., 2015** [38]	N 56; EDSS:0–6; Age 52.9	Exergaming	GSTP, MSW-12, WHODAS2.0
	MS type: NR		
**Garret et al., 2013** [39]	N 121; GNDRS:0,1–2; Age50.05	Circuit exercises, aerobic exercise, yoga	FIS, GNDS,6MWT, MSIS-29
	MS type: RR65, SP20, PP13, Bening 5 Unknown 18		
**Gandolfi et al., 2014** [40]	N 22; EDSS:4.1; Age 50.4	Robot assisted gait training	GSTP, FSS, BBS, SOT, SA, MSQoL-54, ABC
	MS type: NR		
**Peruzzi et al., 2017** [41]	N 25; EDSS:3.8; Age 42.8	Virtual reality and treadmill	ROM, Kinematics, TUG, EDSS, FSST, BBS,6MWT
	MS type: NR		
**Shahraki et al., 2017** [42]	N 18; EDSS:3–6.0; Age 39.2	Gait training/ rhythmic auditory stimulation	GSTP
	MS type: NR		
**Braendvik et al., 2016** [43]	N 29; EDSS:3.2; Age 47.9	Treadmill training	GSTP, Ac
	MS type RR19 SP2 PP5		
**Straudi et al., 2019** [44]	N 72; EDSS:6.5; Age 55.5	Robot assisted gait training	FSS, TUG, T25W, MSWS-12, QoLSF-36, MSIS-29, PHQ-9
	MS type SP38 PP34		
**Heine et al., 2019** [45]	N 10; EDSS:3; Age 48.8	Sequential exercise intervention	GSTP, CPET, MFIS, FMSC, MSWS-12
	MS type RR2 SP3 PP5		
**Callessen et al., 2019** [46]	N 142; EDSS:2–6.5; Age 52	Resistance, Balance, Motor Control training	T25FW, SSST, mBest test, 6MWT, FIS, MS-IM, MSWS-12, CTSIB, ABC
	MS type RR99 SP25 PP18		
**Hochsprung et al., 2020** [47]	N 61; EDSS:2.5–6.5; Age NR	Cycling training with visual feedback	GSTP
	MS type RR27 SP20 PP14		
**Manca et al., 2020** [48]	N 28; EDSS:2.0–5.5; Age 46	High-intensity resistance training	GSTP, ROM, MS-IM
	MS type RR28		
**Mahler et al., 2018** [49]	N 34; EDSS:3.0; Age 50	Robot assisted gait training/	6MWT, 10MWT, Calorimetry, WLT
	MS type RR 34	Overground walking training	
**Mansour 2013** [50]	N 24; EDSS 2.9; Age 40,42	Partial body weight supported treadmill training	TUG, GSTP, Kinematics
	MS type RR 24		
**Manca et al., 2017** [51]	N 30; EDSS:3.4; Age 45.1	Strength training	2MWT, 6MWT, 10MWT, TUG, MS-IM
	MS type RR30		
**Felippe et al., 2019** [52]	N 28; EDSS3.0; Age 50	Treadmill training	TUG, MMSE, FAB
	MS type RR 28		
**McGibbon et al., 2018** [53]	N 29; EDSS:5.2; Age NR	Robot assisted gait training	6MWT, TUG, TST, Ac
	MS type NR		
**Munari et al.,2020** [54]	N 17; EDSS:5.2; Age 54.35	Robot assisted gait training, Virtual reality	PASAT, 2MWT, MSQoL-54, 10MWT, GSTP
	MS type RR 4 SP 14		
**Flachenecker et al, 2020** [55]	N 84; EDSS:4.1; Age 47	Internet-based exercise program	WEI-MuS, MSIS-29, 2MWT, 10MWT, TBS
	MS type RR 39 SP45		
**Elwishly et al.,2020** [56]	N 29; EDSS: 4–6; Age 33.1	Dual task training	2MWT, 10mWT, MMSE, EDSS
	MS type RR 29		
**Esnouf et al., 2010** [57]	N 53; EDSS:5.6; Age 55	FES	COMP, FD
	MS type RR 53		
**Feys et al., 2019** [58]	N 35; EDSS:NR; Age 40.5	Start to run program	VO2, STST, 6MWT, MSFCS, FSMCF, MSIS-29, DTI
	MS type NR		
**Gutierrez et al., 2020** [59]	N 31; EDSS: 3.7; Age 43.5	Strength and dual task combined training program	MS-SS, STS, SA, GSTP
	MS type NR		
**Tollar et al., 2020** [60]	N 68; EDSS:5–6; Age	Exercise therapy	MSIS-29, QoLEQ-5D, TBS, BBS, 6MWT, SA
	MS type		
**Vedkamp et al., 2019** [61]	N 40; EDSS: 3.5; Age 40	Dual task training	T25WT, TUG, DGI, 2MWT, MSWS-12, FES, MSIS-29
	MS type		
**Kahraman et al, 2020**[62]	N 35; EDSS: 1.5; Age 35.2	Motor imagery	DGI, T25WT, 2MWT, MSWS-12, TUG, ABC, MFIS, HADS, SA, SDMT, SRT
	MS type NR		
**Renfrew et al., 2018** [63]	N 78; EDSS: 5.0; Age 39	Electrical stimulation	5MWT, VO2, 25FWT
	MS type RR35 SP18 PP13 NR 12		
**Duff et al., 2018** [64]	N 30; EDSS:NR, PDDS: 2.2; Age 45.5	Pilates	6MWT, TUG, MSQoL-54, FBS, SRT, Ac, MVC
	MS type RR 25, SP 2, PP 3		

Abbreviations: N Number of participants; EDSS Expanded disability status scale; PDDS Patient determined disease steps; NR Not reported; TUG Timed up and go test; 2MWT 2 minute walking test; T25W Timed 25 foot walk; FSST Four square step test; MSWS-12 Multiple sclerosis walking scale-12; MSIS-29 Multiple sclerosis impact Scale_29; ABC Activities-specific balance confidence scale; TBS Tinetti balance scale; HRSD Hamilton rating scale for depression; GSTP Gait spatiotemporal parameters; VO2 oxygen peak uptake; MS-IM Muscle strength isokinetik measures; 6MWT 6-minute walking test; MAS Modified Ashworth scale; MS-MD Muscle strength mechanical device; COPE Coping orientation to problem experienced; MS Type Multiple sclerosis type; RR Remittent recurrent; SP Secondary progressive; PP Primary progressive; ROM Range of movement; VAS visual Analogical Scale; FAC Functional ambulatory scale; FIS Fatigue impact scale; FSS Fatigue severity scale; BBS Berg balance scale; 10m 10-meter walking test; QoL SF-36 Quality of life Short form 36; PHQ-9 Patient health questionnaire; DGI Dynamic gait index; HADS Anxiety and depression scale; MusiQoL Multiple Sclerosis International Quality of Life scale; FRT functional reach test; Rand-36 Random 36 health survey; 3MWT 3-minute walking tests; Ac Accelerometer; WE Wurzburger inventory; MSSS-88 Multiple sclerosis spasticity scale– 88; WHOQoL-bref WHO quality of life bref; AI Ambulatory index; MSIS-29Bref Multiple sclerosis impact scale_29; MRC Medical research council; BDI Beck depression inventory; WHODAS2.0 World health organization disability assessment schedule; GNDS Guy’s neurological disability scale; SOT Sensory organization test; SA Stabilometric assessment; CPET Maximal cardiopulmonary exercise test; FMSC Fatigue scale for motor and cognitive function; SSST Six spot step test; WLT Working load support; MMSE Mini mental state examination; FAB Frontal assessment battery; TST Timed stair test; PASAT Paced auditory serial attention test; Wei-MuS Wurzburger fatigue inventory; COPM Canadian occupational performance measure; FD Falls diary; STS Sit to stand test; MSFCS Multiple sclerosis functional composite; MS-SS Muscle strength static strength; QoL EG-D Quality of life questionnaire; SRT Sit and reach test; 5MWT 5 minute walking test; FBS Fullerton balance scale.

**Table 2:** Outcome measures found in included RCTs and their abbreviations.

**10 m**	10-meter walking test	MRC	Medical research council
**2MWT**	2-minute walking test	**MSFCS**	Multiple sclerosis functional composite
**3MWT**	3-minute walking test	**MS-IM**	Muscle strength isokinetik measures
**5WWT**	5-minute walking test	**MSIS_29v2**	Multiple sclerosis impact scale_29
**6MWT**	6-minute walking test	**MS-LD**	Muscle strength lokomat device
**ABC**	Activities-specific balance confidence scale	**MS-MD**	Muscle strength mechanical device
**Ac**	Accelerometers	**MSQoL-54**	Multiple sclerosis quality of life
**AI**	Ambulatory index	**MSSE**	Mini metal state examination
**BBS**	Berg balance scale	**MS-SS**	Muscle strength static strength
**BDI**	Beck depression inventory	**MSSS-88**	Multiple sclerosis spasticity scale—87
**Calorimetry**		**MSWS-12**	Multiple sclerosis walking scale-11
**COPM**	Canadian occupational performance measure	**MusiQoL**	Multiple sclerosis international quality of life scale
**COPE**	Coping orientation to problem experienced	**PASAT**	Paced auditory serial attention test
**CPET**	Maximal cardiopulmonary exercise test	**PDDS**	Patient determined disease steps
**CTSIB**	Test for sensory interaction and balance	**PHQ-9**	Health questionnaire
**DGI**	Dynamic gait index	**QoL EQ-D**	Health questionnaire
**DTI**	Diffusor tensor imaging	**QoL SF-36**	Quality of life short form 36
**EDSS**	Expanded disability status scale	**RAND—36**	Rand 36 health survey
**FAB**	Frontal assessment battery	**ROM**	Range of movement
**FAC**	Functional ambulatory scale	**SA**	Stabilimetric assessment
**FBS**	Fullerton balance scale	**SOT**	Sensory organization test
**FD**	Falls diary	**SRT**	Sit and reach test
**FES**	Falls efficacy scale	**SSST**	Six spot step tests
**FIS/mFIS**	Fatigue impact scale	**STS**	Sit to stand tests
**FMSC**	Fatigue scale for motor and cognitive function	**T25W**	Timed 25-foot walk
**FRT**	Functional reach test	**TBS**	Tinetti balance scale
**FSS**	Fatigue severity scale	**TST**	Timed stair test
**FSST**	Four square step test	**TUG**	Timed up and go
**Kinematics**		**VAS (pain)**	Visual analogic scale (pain)
**GNDS**	Guy’s neurological disability scale	**VO2**	Oxygen peak uptake
**GSTP**	Gait spatio temporal parameters	**WEI-MuS**	Wurzburger fatigue inventory
**HADS**	Anxiety and depression scale	**WHODAS 2.0**	World health organization disability assessment schedule
**HRSD**	Hamilton rating scale for depression	**WHOQoL-Bref**	WHO quality of life-bref
**MAS**	Modified Ashworth scale	**WLT**	Working load treadmill
**MiniBestTest**	Mini best test		

### Most commonly used outcome measures according to ICF levels

**Fig 2:**
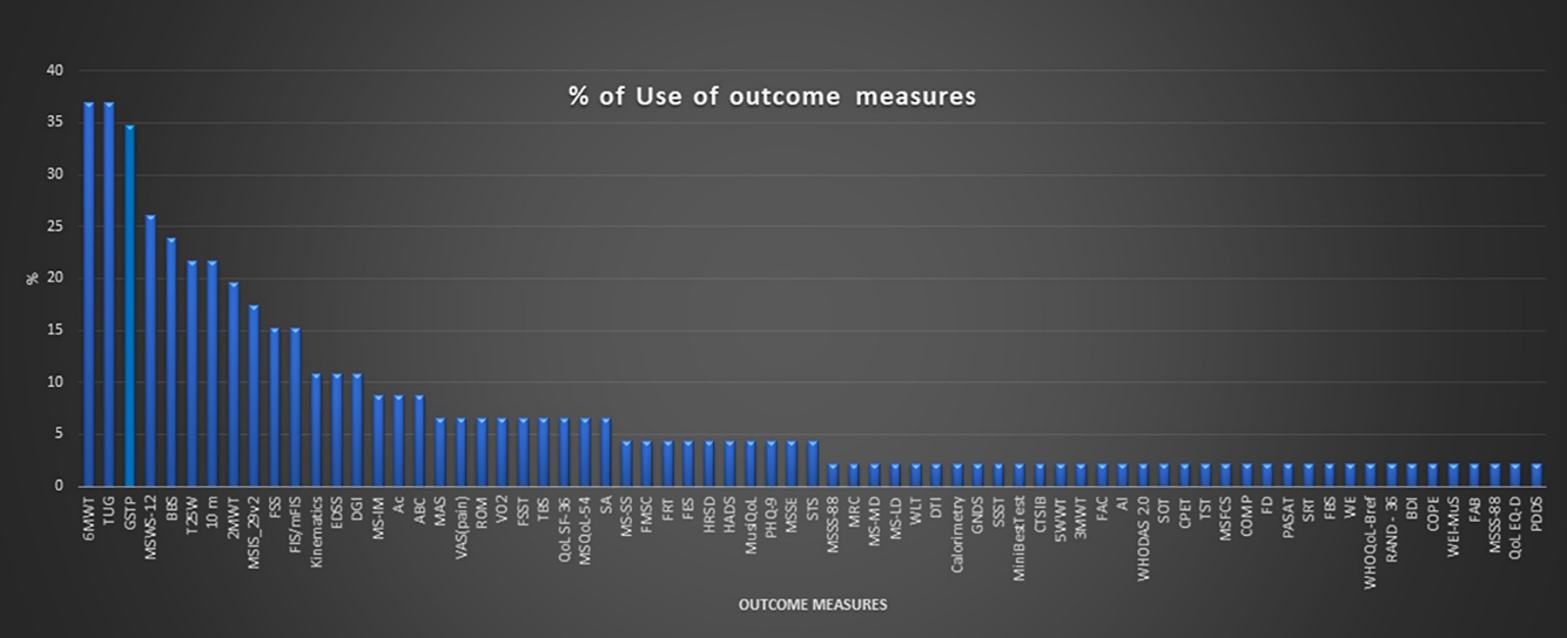
Graph showing the percentage of use of each outcome measure. Abbreviations: 6MWT 6-minute walking test; TUG Timed up and go test; GSTP Gait spatio-temporal parameters; MSWS-12 Multiple sclerosis walking scale-12; BBS Berg balance scale; T25W Timed 25-foot walk; 10m 10-meter walking test; 2MWT 2-minute walking test; MSIS-29 Multiple sclerosis Impact Scale_29; FSS Fatigue severity scale; FIS/mFIS Fatigue impact scale/modified fatigue impact scale; EDSS Expanded disability status scale; DGI Dynamic gait index; MS-IM Muscle strength isokinetik measure; Ac Accelerometers; ABC Activities-specific balance confidence scale; MAS Modified Ashworth scale; VAS (pain) Visual analogic scale (pain); ROM Range of motion; VO2 peak oxygen uptake; FSST Four step square test; TBS Tinetti balance scale; QoL SF-36 Quality of life short form 36; MSQoL-54 Multiple sclerosis quality of life; SA Stabilometric assessment; MS-SS: Muscle strength static strength; FMSC Fatigue scale for motor and cognitive function; FRT Functional reach test; FES Falls efficacy scale; HRSD Hamilton rating scale for depression; HADS Anxiety and depression scale; MusiQoL Multiple Sclerosis International Quality of Life scale; PQH-9 Patient health questionnaire; MSSE Mini mental state examination; STS Sit to stand test; MSSS-88 Multiple sclerosis Spasticity Scale– 88; MRC Medical research council; MS-MD Muscle strength mechanical device; MS-LD Muscle strength lokomat device; WLT Working load in treadmill; DTI Diffusion tensor imaging; GNDS Guy’s Neurological Disability Scale; SSST Six spot step tests; mBEest Test Mini best test; CTSIB Test for sensory interaction and balance; 5MWT 5-minute walking tests; 3MWT 3-minute walking test; FAC Functional ambulatory scale; AI Ambulatory index; WHODAS 2.0 World health organization disability assessment schedule; SOT Sensory organization test; CPET Maximal cardiopulmonary exercise test; TST Timed stair test; MSFCS Multiple sclerosis functional composite; COPM Canadian occupational performance measure; FD Falls diary; PASAT Paced auditory serial attention test; SRT sit and reach test; FBS Fullerton balance scale; WE Wurzburger inventory; WHOQoL-Bref WHO quality of life-bref; RAND-36 Random 36 health survey; BDI Beck depression inventory; COPE Coping Orientation to Problem Experienced; WEI-MuS Wurzburg Fatigue Inventory for Multiple Sclerosis; FAB Frontal assessment battery; MSSS-88 Multiple sclerosis Spasticity Scale; QoL-EQD Euro quality of life; PDDS Patient Determined Disease Steps.

The wide range of outcome measures used across RCTs is depicted in Fig 2. The most used outcome measures were the 6-minute walking test and the Timed Up and Go test, followed by gait spatiotemporal parameters (GSTP).

**Fig 3:**
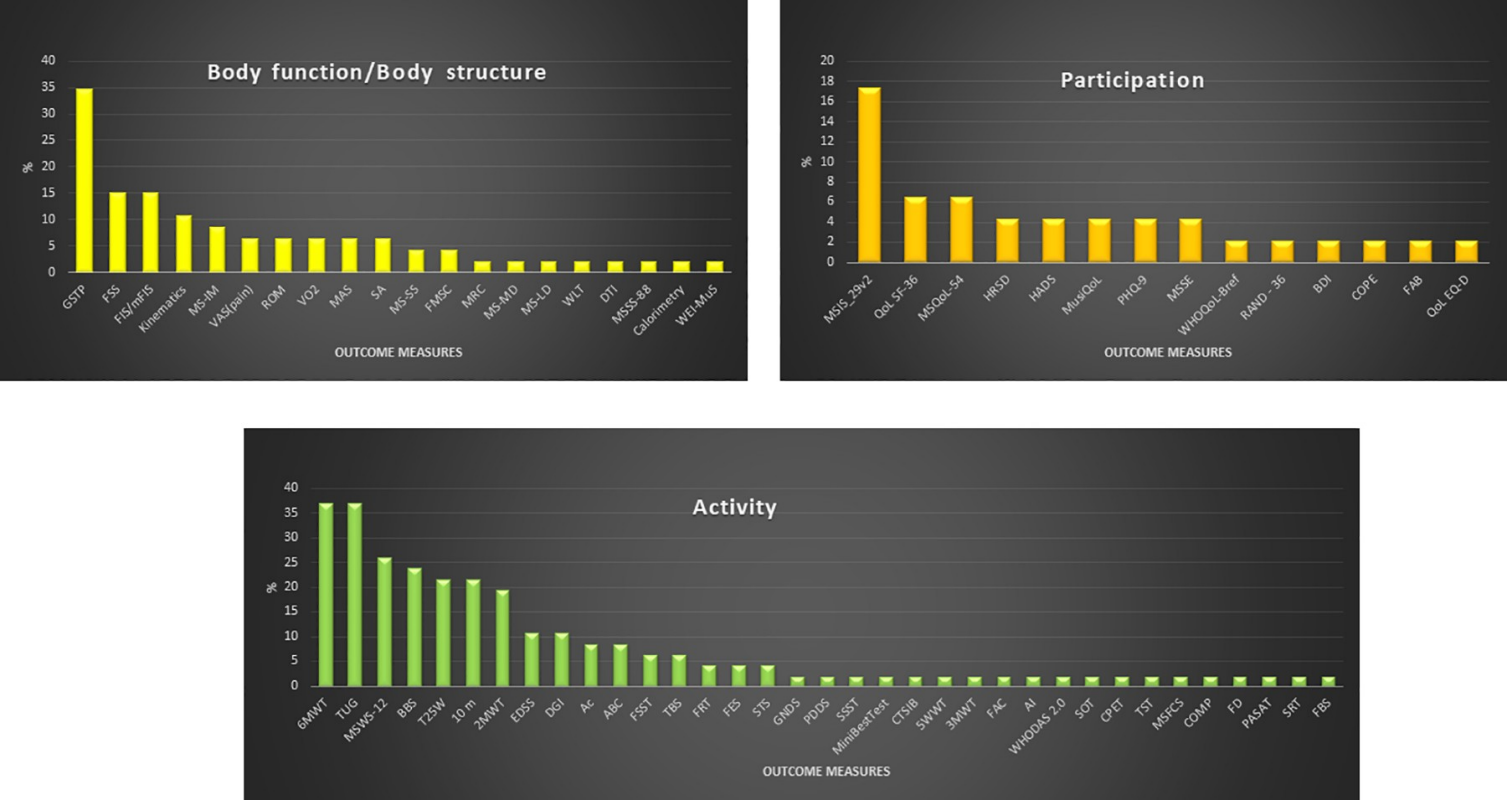
Outcome measures used according to ICF (% of use). Abbreviations: GSTP Gait spatiotemporal parameters; FSS Fatigue severity scale; FIS/mFIS Fatigue impact scale/modified fatigue impact scale; MS-IM Muscle strength isokinetik measure; VAS (pain) Visual analogic scale (pain); ROM Range of motion; VO2 peak oxygen uptake; MAS Modified Ashworth scale; SA Stabilometric assessment; MS-SS: Muscle strength static strength; MSC Fatigue scale for motor and cognitive function; MRC Medical research council; MS-MD Muscle strength mechanical device; MS-LD Muscle strength lokomat device; WLT Working load in treadmill; DTI Diffusion tensor imaging; MSSS-88 Multiple sclerosis Spasticity Scale– 88; WEI-MuS Wurzburg Fatigue Inventory for Multiple Sclerosis; MSIS-29 Multiple sclerosis Impact Scale_29; QoL SF-36 Quality of life short form 36; MSQoL-54 Multiple sclerosis quality of life; HRSD Hamilton rating scale for depression; HADS Anxiety and depression scale; MusiQoL Multiple Sclerosis International Quality of Life scale; PQH-9 Patient health questionnaire; MSSE Mini mental state examination; WHOQoL-Bref WHO quality of life-bref; RAND-36 Random 36 health survey; BDI Beck depression inventory; COPE Coping Orientation to Problem Experienced; FAB Frontal assessment battery; QoL-EQD Euro quality of life; 6MWT 6-minute walking test; TUG Timed up and go test; MSWS-12 Multiple sclerosis walking scale-12; BBS Berg balance scale; T25W Timed 25-foot walk; 10m 10-meter walking test; 2MWT 2-minute walking test; EDSS Expanded disability status scale; DGI Dynamic gait index; Ac Accelerometers; ABC Activities-specific balance confidence scale; FSST Four step square test; TBS Tinetti balance scale; FRT Functional reach test; FES Falls efficacy scale; STS Sit to stand test; GNDS Guy’s Neurological Disability Scale; PDDS Patient Determined Disease Steps; SSST Six spot step tests; Mini Best Test Mini best test; CTSIB Test for sensory interaction and balance; 5MWT 5-minute walking tests; 3MWT 3-minute walking test; FAC Functional ambulatory scale; AI Ambulatory index; WHODAS 2.0 World health organization disability assessment Schedule; SOT Sensory organization test; CPET Maximal cardiopulmonary exercise test; TST Timed stair test; MSFCS Multiple sclerosis functional composite; COPM Canadian occupational performance measure; FD Falls diary; PASAT Paced auditory serial attention test; SRT sit and reach test; FBS Fullerton balance scale.

Of the 69 outcome measures found, 20 assessed *Body function and Body structure*, 35 assessed *Activity* and 14 assessed *Participation* domains of ICF (See Fig 3). 17% of the studies assessed only one ICF domain, 44% of RCTs included measures covering two ICF domains and only 39% measures from all three ICF domains.

The *Body structure/Body function* domain was assessed in 80% of studies and the most used outcome measure to assess this domain was GSTP, used in 35% of RCTs. GSTP referred to b770 on ICF domain [15], was performed using different systems: nine studies used the Gaitrite system, two used the Vicon system, one used the Smart-D BTS bioengineering system, two used the Qualisys motion system, one study used the Gait-Real-time-Analysis-Interactive-Lab and one study a 3D photogrammetry. All these systems provide GSTP and some of these technological systems provide kinematics parameters with information about displacement and range of movement of joints. In studied RCT only 10% provide kinematic parameters.

In terms of GSTP, most studies (87%) reported gait speed, 67% of these studies reported cadence (steps/minute), 56% reported step length, and 37% analyzed stride length. Specific GSTP used in each study are reported in Table 3.

**Table 3:** Gait spatiotemporal parameters.

	AS	FAP	GS	Cd	StT	SuT	St L	Sd L	Others
**Sandroff 2017**	GAITRite	X	X	X	X		X		
**Pau 2017**	BTS Bioengieniering		X	X		X		X	GPS
**Davies 2016**	GAITRite		X	X			X		
**Ruiz 2013**	GAITRite		X	X					
**Conklin 2010**	GAITRite	X	X	X		X	X	X	
**Robinson 2015**	GAITRite	X	X	X			X	X	HHBS
**Gandolfi 2014**	GAITRite		X	X		X	X		
**Peruzzi 2016**	VICON		X				X	X	
**Shahraki 2017**	QMA		X	X		X		X	Stride time
**Braendvik 2015**	GAITRite	X							ARMS, VMD
**Manca 2020**	VICON		X	X					
**Hochsprung 2020**	GAITRite	X	X	X					
**Heine 2019**	GRAIL					X	X		SW
**Mansour 2013**	QMA			X				X	
**Munari 2020**	GAITRite		X			X	X		HHBS, SSP
**Gutierrez 2020**	3D photogrametry		X			X	X		

Abbreviations: AS Assessment system; FAP Functional ambulatory Profile (GAITRite specific); GS Gait speed; Cd Cadence; St T Step time; Su T Support time; St L Step length; Sd L Stride length; GPS Gait Profile Score; HHBS Hell to hell base support; QMA Qualysis motion analysis; ARMS Acceleration root mean square; VMD Vertical and mediolateral direction; GRAIL Gait-Real-time-Analysis-Interactive-Lab; SW Step width; SSP Stance and swing phase.

*Fatigue*, referred by the Body function/Body structure ICF item b4552 [15], is a cardinal symptom in MS impacting on gait pattern and functioning, and was assessed in 39% of studies using four different scales, the fatigue severity scale (15% of studies), the fatigue impact scale (15% of studies), the fatigue scale for motor and cognitive function (4% of studies), and the Wei-MUS scale (4% of studies).

*The Activity* domain was assessed in 91% of studies, assessing walking capacities referring to d450 ICF item (walking) and d4609 item (move around) [15]. Following the 6-minute walking test and the Timed Up and Go test used in 37% of studies, the Multiple Sclerosis Walking Scale-12 was used in 26% of studies and the Berg Balance Scale was used in 24% of studies. The expanded disability status scale (EDSS) for MS is used in 91% of the studies. Studies used the EDSS for different purposes. Only 13.33% used the EDSS to assess intervention efficacy and 80% of the studies used EDSS for classifying clinical status of the participants.

*Participation* and quality of life was assessed in 50% of studies, using 14 different scales. The most used outcome measure to assess this domain was the Multiple Sclerosis Impact Scale 29, used in 17% of the studies, followed by the Quality of Life Short Form 36, used in 6% of the studies.

How outcome measures are distributed according to ICF levels is described in Fig 3.

### Combination of outcome measures

**Fig 4:**
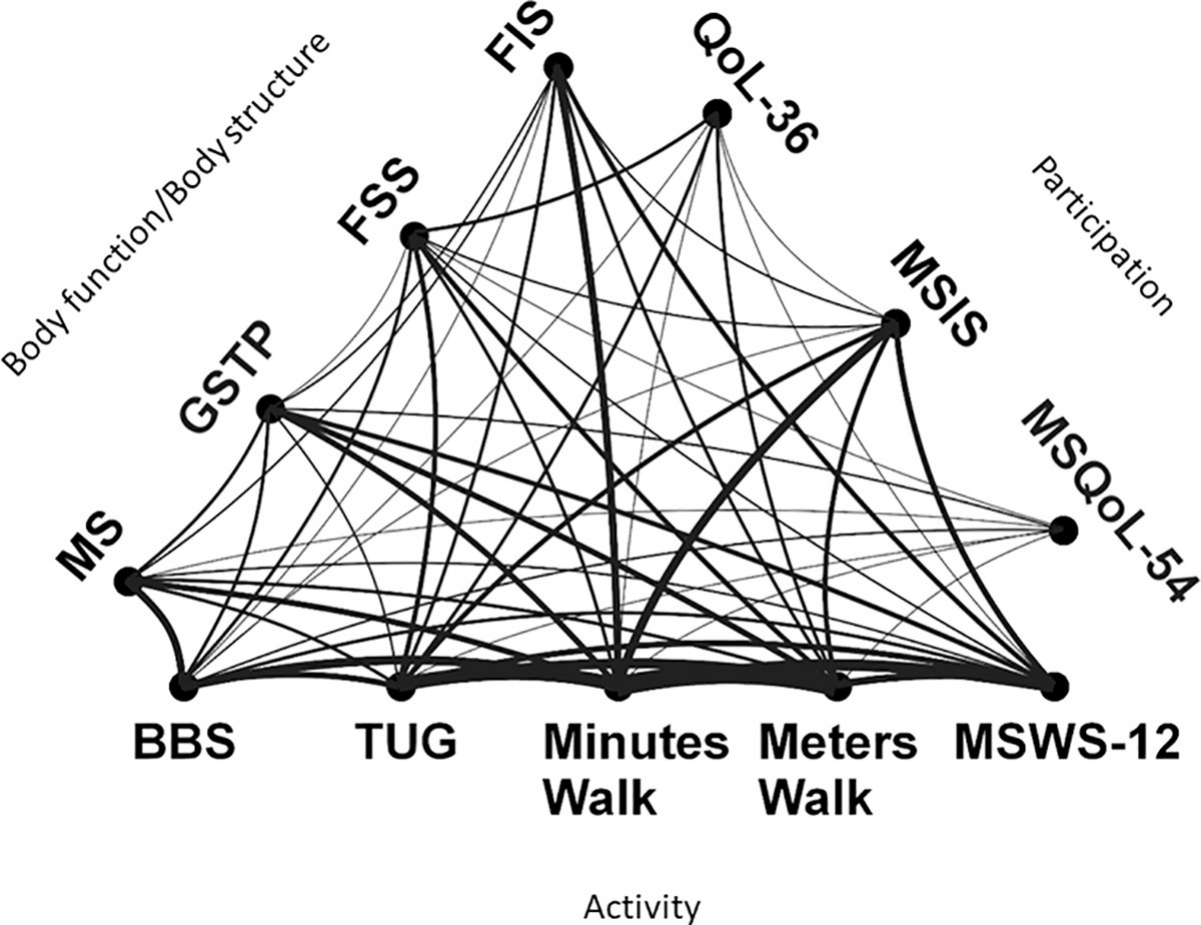
How outcome measures were combined in studies. Abbreviations: FSS Fatigue Severity Scale; FIS Fatigue impact scale; GSTP Gait spatio temporal parameters; Ms Muscle strength; MSIS Multiple sclerosis impact scale; QoLSF- 36 Quality of life short form 36; MSQoL-54 Multiple sclerosis quality of life 54; MSWS-12 Multiple Sclerosis Walking Scale-12; Minute walk 2-minute,3-minute, 5-minute and 6-minute walking Tests merged; Meters walk 10-meter walking test/Timed 25-foot walk test merged; TUG Timed Up and Go; BBS Berg Balance Scale.

How often outcome measures were combined with each other is shown in Fig 4. Four scales were combined as ‘Minutes walked’: 2-meter walking test, 3-minute walking test, 5-minute walking test, and 6-minute walking test. ‘Meters walked’ represents a combination of 10-meter walking test and the Timed 25-foot walk test. Ms represents combination of muscle strength with Lokomat device, isokinetic dynamometers, mechanical devices, and static strength measures.

The most common combination of measures was between ‘Meters walked’ and ‘Minutes walked’ measures used in 32% of studies (15 RCT) and between ‘Minutes walked’ and Timed Up and Go used in 24% of studies (11 RCT).

The most common inter-domain combinations of measures were between Fatigue Impact Scale on *Body structure/Function* level and ‘Minutes walked’ measure on *Activity* level (85% of studies using FIS) and between Multiple Sclerosis Impact Scale on *Participation* level and ‘Minutes Walked’ (88% of studies using MSIS) on *Activity* level.

GSTP assessment was complemented by other clinical mobility measures: 31% of them used a measure of *walking time* (predominantly 6-minute walking test) and 31% of studies also assessed Timed 25-foot walk test (*meters walked*; Fig 4). GSTP was less often combined with Berg Balance Scale (three studies, 19%) and Multiple Sclerosis Walking Scale-12 (four studies, 25%) and Timed Up and Go (two studies, 12%). GSTP was combined with muscle strength measurement in 19% of studies, but was rarely combined with fatigue measures (only one study, 6%) using Fatigue Severity Scale, and was combined with quality of life or participation assessments in only two RCT.

In Fig 4. we can see how outcome measures were combined in studies. Represented by a line between scales, the thicker the line is the more often the two scales are used in the same RCTs.

### Outcome measure selection adapted to severity of MS

We stratified studies according to clinical status and gait capacity of the participants to study whether this influenced selection of outcome measures. A score of 4.5 on EDSS has been used [65, 66] to classify MS participants into those with mild walking disability (score <4.5) and moderate to severe (score >4.5) gait disturbance [67]. In 19 RCTs, including participants with severe gait disturbance according to EDSS, the Timed Up and Go was the most used outcome measure, used in 47% of studies, followed by the 6-minute walking test used in 42% of studies. In 22 RCTs with less affected participants, the most used outcome measure was GSTP used in 54% of studies followed by the 6-minute walking test and Multiple sclerosis walking scale-12, used in 32% of studies.

## 4. Discussion

This systematic review showed that the most used outcome measures in RCTs on gait interventions in MS were the 6-minute walking test and the Timed Up and Go test, followed by GSTP, and that the choice of outcome measures depended on MS disease severity of participants. This study also highlights the large heterogeneity in the outcome measures used, and the fact that only the 39% of analyzed studies considered the three ICF domains in their assessment.

### Gait spatiotemporal parameters and clinical assessments of gait

Assessments performed with technological devices to assess GSTP provide clinicians and researchers with accurate objective information. The studied parameters included time or distance parameters like stance duration, swing duration, stride length, gait cycle duration, cadence, velocity and normalized velocity [68]. One advantage of technological gait evaluation is that specific and sensitive information about gait quality (e.g., lower limb movement symmetry, support phase symmetry) and gait pattern (e.g., spastic-paretic, ataxia like, unstable gait) [69] is obtained allowing to gauge the impact of the studied interventions on these aspects.

In reviewed studies, the GSTP most often assessed with technological devices was gait speed. Other parameters like step length or support are not sensitive enough to detect changes in gait capacity across EDSS spectrum of mobility [70].

In the included RCTs, GSTP were more frequently reported in studies on patients with mild EDSS (score <4.5). GSTP were also often combined with clinical assessment of gait, mobility, and balance (6-minute walking test, Timed Up and Go, Berg Balance Scale; see Fig 4). Included RCTs have thus provided comprehensive evaluations of gait.

There is a growing tendency to use GSTP to assess gait capacities in RCTs. Despite this fact, studies on the psychometric properties of these methods is needed. This point was already pointed out by Andreopoulou in 2019 [71], stating that although 3D gait analysis is considered a “gold” standard, psychometric properties of some of the measures provided by these technological systems have not been examined in pwMS. They studied the relative and absolute reliability of ankle kinematics and GSTP provided by VICON system in a sample of 49 pwMS. Their results indicate good to excellent relative reliability of walking speed, step length and cadence. Psychometric properties of other systems like GAITrite have been studied. Riis in 2020 [72] studied its convergent validity in a sample of 24 geriatric patients, studying correlations between Berg Balance Scale, DGI and Timed Up and Go test, showing moderate correlations between GAITrite parameters and functional tests. Hoschproung in 2014 [73] compared GAITrite provided GSTP with results of the Timed 25-foot walk test in a sample of 85 pwMS, obtaining as results that the GAITrite system has the same clinical validity in gait evaluation as the Timed 25-foot walk test. Sosnoff in 2011 [74] studied the validity of the functional ambulatory profile (FAP) score from GAITrite in a sample of 13 pwMS. They found that this specific parameter strongly correlated with the EDSS, walking performance (Timed 25-foot walk tests and Timed Up and Go tests) supporting validity of this GAITrite measure. But there is still a lack of knowledge about psychometric properties of GSTP obtained using other technological systems.

The most used clinical scales for gait assessment in the Activity domain of the ICF were the following: 6-minute walking test, Timed Up and Go test, 10-meter walking test, Timed 25-foot walk test. These clinical measures have good psychometric properties [75] and they assess gait in a quantitative manner. The 6-minute walking test gives information about cardiopulmonary function, and also provides information about walking capacities; the Timed Up and Go test provides quantitative information about gait and functional capacities, assessing a sit to stand transfer from a chair followed by 3 meter walk, a turning and a return to the sitting position, allowing to assess also dynamic balance and gait stability; the Timed 25-foot walk test is a short distance measure of walking speed; the 10-meter walking test assesses a short distance walk allowing to asses gait speed [76]. All these tests can be complementary to each other, giving information about different aspects of gait. But it is difficult to compare efficacy of interventions across RCTs when different outcome measures are used. This makes clinical decision making and the establishment of evidence-based guidelines challenging, particularly when metanalyses are lacking.

### Gait speed

Gait speed was the most commonly used GSTP and was also measured in clinical gait assessments. There is thus good consensus among clinical researchers to use gait speed to assess efficacy of gait rehabilitation interventions. There are other authors that describe gait speed as a suitable outcome to assess differences in gait performance [70]. However, GSTP, 10-meter walking test, 2-minute walking test, 3-minute walking tests, and the Timed 25-foot walk, assess gait speed in different ways. Gait speed over short distances is assessed in the 10-meter walking test, and Timed 25-foot walk test, while 2-minute walking test, 3-minute walking test, 5-minute walking test, and 6-minute walking test assess gait speed and endurance over longer distances. Clinical scales and assessment with technological systems also differ in terms of instructions provided to the subject or required speed (maximal speed, comfort speed), with no standardized protocol for every technological system.

Gait speed seems to be the parameter that researchers choose to assess gait rehabilitation interventions, assessing gait capacities in a quantitative manner. Although all trials include gait speed as an outcome measure, it is difficult to compare across clinical trials since testing procedures differed, e.g., distances covered and instructions provided were not the same. A consensus about modalities of assessment of this parameter, including standardized protocol for short and long-distance testing, could help in comparing results across RCTs.

Although gait speed is one of the parameters that is affected in pMS, decreasing while EDSS increases [69], one may ask if improving gait speed in performed tests really reflects an improvement in gait capacities. A less studied aspect, walking speed reserve (i.e., the difference between usual and fastest speed) could be important for interpretation of RCT results. Gijbels in 2010 [77] found that pace instructions provided influenced gait speed of the participants. They also reported that the difference between comfortable self-induced walking pace and fastest possible walking speed decreases as the degree of ambulatory dysfunction increases. That means that in more affected patients the performed gait speed is not necessarily a reflection of their comfortable walking speed. Taking this discrepancy into account in RCTs on gait interventions could help in improving accuracy and identifying efficacy of interventions on gait capacities.

### Fatigue

Fatigue is a cornerstone symptom in pwMS [78] that likely determines gait pattern and gait functionality in everyday life [79, 80]. In our results we can see that 39% of studies assessed this aspect using four different scales. To know which gait rehabilitation intervention minimizes this symptom is central for optimal clinical decision making.

Few studies combined GSTP evaluation with measures of fatigue. This highlights a gap in previous research priorities in RCTs on gait interventions. Fatigue interacts with GSTP, for example, fatigue can be reflected by changes in stride length, gait velocity and stride time [81]. Future RCTs should therefore combine GSTP and fatigue measurements for a more complete mechanistic understanding.

### Participation

Reducing restriction in Participation and obtaining good quality of life is the overall objective of rehabilitation interventions. Quality of life questionnaires provide useful information about this aspect that is identified by therapists as one of the goals of their therapies [82]. However, Participation was not systematically assessed (only 50% of studies assessed it) and there was considerable heterogeneity in the choice of outcome measures, with 14 different outcome measures for assessing Participation. Assessing this aspect more frequently in RCTs on gait interventions is recommended since this review showed a lack of consensus among researchers on the need to assess this aspect and on which measure to select. Improved consensus here would make it possible to compare the effects of rehabilitation interventions on quality of life across studies more easily.

In our findings, GSTP were combined with Participation assessments in only two studies, showing that most RCTs that focus on objective and fine assessment of gait parameters do not consider the repercussion of the studied intervention on the patient’s specific life context. It is important that future studies on gait interventions combine these measures to extend results on pwMS quality of life, which is the final objective of rehabilitation interventions and enable more comprehensive understanding of intervention effects.

### Gait capacities characterized by EDSS

EDSS is widely used for defining participant characteristics [65, 66, 83] and in our results, we observed that different outcome measures were used depending on gait capacities assessed by the EDSS.

Assessment with EDSS have many limitations [84], and assessments capable of compensating these limitations are needed when assessing gait capacities. Some outcome measures can be challenging for patients with a high EDSS, while others may not be sensitive enough to assess changes in pwMS with high gait capacities. GSTP, for example, were more frequently used in less affected pwMS characterized with a lower EDSS that need a fine assessment to detect changes in gait, since other tests like Timed Up and Go test can have ceiling effects and would not be responsive enough to changes due to rehabilitation interventions. In contrast, Timed Up and Go test, which provides information about gait over short distances and functional aspects like transfers, was used in more affected patients with higher scores in EDSS.

Regarding GSTP in pwMS, absolute and relative reliability of GSTP have been studied [71] in populations with lower (0–3.5) and higher (4–6) EDSS scores, and this study showed that higher walking disability in pwMS was associated with higher within-subject variability. These results are consistent with our review findings showing that clinical researchers less often chose this kind of assessment in pwMS with lower gait capacities.

### Measuring across ICF domains

Comprehensive assessment, with outcome measures spanning all the *ICF domains*, is counseled by European recommendations in MS rehabilitation (RIMS) [16], and International Consensus Conference about ICF core sets in MS [15]. A recent study about goal setting and assessment according to ICF in MS, points out the need to use ICF Core Sets and standardized outcome measures for evaluation at the different ICF levels, both in clinical practice and in research (82). This multidimensional assessment can give information about efficacy of gait interventions on the global status of the pwMS and not only about one specific component. As we can see in our results, only 39% of analyzed clinical trials consider the three domains of the ICF. Covering all ICF domains more systematically in studies will be useful for comparing the global efficacy of physical interventions among studies. Combining Participation measures with GSTP would allow to answer whether gait interventions that improve quality of gait also enhance quality of life of pwMS. The assessment using the ICF framework has also been recommended in other neurological diseases like Parkinson’s [85], stroke [86] and also in pediatric pathology [87].

There are some authors that have already pointed out the need to refine the assessment in MS clinical trials, alluding to the need for multidimensional measures in order to allow full coverage of disease progression and the value of technological measures [10, 80]. Nonetheless, our results point to a lack of consensus among researchers as to the best outcome measures to assess gait performance in all ICF domains after gait rehabilitation interventions in MS.

### Implications for research

There are literature reviews about measurement properties of gait assessment in people with MS [88], and some authors have been interested in studying psychometric properties of specific technological devices for assessment in MS [11]. However, there is still a lack of knowledge of psychometric properties of all technological devices used to assess GSTP in pwMS.

There is a clear need for a systematic review evaluating measurement properties of gait assessment in people with MS, including all technological systems used for assessing GSTP, to recommend specific outcome measures for future studies.

### Limitations of the study

In this review we only included RCTs. Data from longitudinal or cross-sectional studies was not included.

We have analyzed the influence of gait capacities on the choice of outcome measures, but we have not analyzed whether the type of MS can influence this choice.

Neither have we analyzed whether the sample of participants in studies could influence the choice of outcome measures.

Another limitation is that we have only included studies on rehabilitation interventions if the aim of the study was to improve gait capacities. There are rehabilitation interventions like balance interventions, vestibular specific interventions or exercise interventions that focus on improving specific aspects other than gait capacities, which can have an influence in gait performance, that are not included in this review.

## 5. Conclusion

Assessment in pwMS poses a great challenge due to the heterogeneity of symptoms and the progressive changing status of pwMS. This systematic literature review highlights the heterogeneity in choice of outcome measures used in RCTs on gait interventions and the lack of systematic assessment across the whole ICF spectrum. Improved consensus in assessment across studies would help clinicians and researchers interpret results of rehabilitation interventions and facilitate meta-analyses to compare results across studies [18]. Assessment of the whole ICF spectrum is needed to determine which gait interventions are the most efficient ones to improve capacities at Body structure and Body function, Activity, and Participation levels. A growing consensus was identified for the use of GSTP to evaluate the effects of gait interventions. These measures were often combined with clinical gait, mobility, and balance measures. However, GSTP were rarely combined with measures of fatigue or Participation, highlighting an important gap in research knowledge. Continued efforts are needed to move forward in establishing consensus on selection of outcome measures in clinical trials on gait interventions in MS and assessing psychometric properties of commonly used assessment methods.

## Supporting information

S1 Checklist Prisma 2009 checklist. (DOC)

S1 Data (XLSX)
