# Correction: Outcome measures used in trials on gait rehabilitation in multiple sclerosis: A systematic literature review

**DOI:** 10.1371/journal.pone.0314427

**Published:** 2024-11-19

**Authors:** L. Santisteban, M. Teremetz, J. Irazusta, P. G. Lindberg, A. Rodriguez-Larrad

There are errors in the author affiliations. The correct affiliations are as follows:

L. Santisteban^1^, M. Teremetz^3^, J. Irazusta^1,2^, P. G. Lindberg^3^, A. Rodriguez-Larrad^1,2^

**1** Department of Physiology, University of the Basque Country, UPV/EHU, Leioa, Spain, **2** Biocruces Bizkaia Health Research Institute, Barakaldo, Bizkaia, Spain, **3** Institute of Psychiatry and Neuroscience of Paris, INSERM U1266, Université Paris Descartes, Sorbonne Paris Cité, Paris, France.
